# Abstract of Proceedings of the Buffalo Medical Association

**Published:** 1868-02

**Authors:** T. M. Johnson

**Affiliations:** Sec’y.


### ART. II.—*Abstract of Proceedings of the Buffalo Medical Association.*

TUESDAY EVENING, January 7th, 1868.

The President and Vice President being absent, Dr. B. T. Whitney was elected Chairman of the meeting. Members present—Drs. Witney, Diehl, Congar, Edmonds, Daggett, Potter, Smith, Samo, Kamerling, Abbott and Johnson.

The minutes of the last meeting were read and approved.

Dr. H. M. Congar read a paper on the "Production of Sex."

DR. SAMO moved a vote of thanks to Dr. Congar for his interesting paper. Carried.

DR. B. T. WHITNEY said that in behalf of his associates in dentistry, he wished to ask the coöperation of this Association in the endeavors now being made to procure the enactment by the present Legislature of this State, of a law to regulate the study and practice of dentistry. The Doctor explained the substance of the law sought for and the necessity of such a law, and at the close of his remarks offered the following resolution, which was unanimously adopted:

*Resolved*, That this Association heartily concur in the efforts now being made to secure the passage of some law by the Legislature that may tend to regulate the study and practice of dentistry and to protect the people of this State from unskillful dental operations.

Scarlatina was reported as prevailing.

Adjourned. T. M. JOHNSON, Sec'y.

---

AVERAGE DURATION OF LIFE IN ITALY.—The director of the Italian Life Assurance Society, M. W. Rey, has published some interesting statistics showing the average duration of life in Italy as compared with that in other countries, from which it appears that the mortality of Italians is exceptionally great. He shows that in Italy, 22½ per cent. of the infant population die yearly, and that, even in the healthiest districts, the average duration of life is 33.43 years only, while in France it is 38.33, at Geneva, 42.02, and in England, 39.31. The number of births, too, is relatively much smaller in Italy than in England and France.

